# Attentional Bias for Cues Signaling Punishment and Reward in Adolescents: Cross-Sectional and Prognostic Associations with Symptoms of Anxiety and Behavioral Disorders

**DOI:** 10.1007/s10802-020-00654-3

**Published:** 2020-05-22

**Authors:** L. J. Kreuze, N. C. Jonker, C. A. Hartman, M. H. Nauta, P. J. de Jong

**Affiliations:** 1 Department of Clinical Psychology and Experimental Psychopathology, University of Groningen, Grote Kruisstraat 2/1, 9712 TS Groningen, The Netherlands; 2 Department of Psychiatry, Interdisciplinary Center Psychopathology and Emotion regulation (ICPE), University of Groningen, University Medical Center Groningen, Groningen, The Netherlands

**Keywords:** Anxiety, Behavioral problems, Attentional bias, Punishment sensitivity, Reward sensitivity, Young people

## Abstract

Heightened reward sensitivity has been proposed as a risk factor for developing behavioral disorders whereas heightened punishment sensitivity has been related to the development of anxiety disorders in youth. Combining a cross-sectional (*n* = 696, mean age = 16.14) and prospective (*n* = 598, mean age = 20.20) approach, this study tested the hypotheses that an attentional bias for punishing cues is involved in the development of anxiety disorders and an attentional bias for rewarding cues in the development of behavioral disorders. A spatial orientation task was used to examine the relation between an attentional bias for punishing cues and an attentional bias for rewarding cues with anxiety and behavioral problems in a subsample of a large prospective population cohort study. Our study indicates that attentional biases to general cues of punishment and reward do not seem to be important risk factors for the development of anxiety or behavioral problems respectively. It might be that attentional biases play a role in the maintenance of psychological problems. This remains open for future research.

**Electronic supplementary material:**

The online version of this article (10.1007/s10802-020-00654-3) contains supplementary material, which is available to authorized users.

## Introduction

Given that developmental pathways are triggered or become rooted during adolescence, mental health problems in adolescence may have long-term consequences (Ormel et al. 2015). The prevalence of psychiatric illnesses rises from childhood to young adulthood (Copeland et al. 2011; Newman et al. 1996). Adolescence and young adulthood especially are periods with increasing demands for coping with stress resulting from the multiple transitions in these periods (Leadbeater et al. 2012). This underscores the importance of understanding mechanisms involved in the development of mental disorders during adolescence and young adulthood. Two prevalent classes of disorders with age-of onset in childhood and adolescence are anxiety disorders and behavioral disorders (Ormel et al. 2015). Two important traits that have often been linked to symptoms of anxiety and behavioral disorders are sensitivity to punishment (PS) and sensitivity to reward (RS).

Punishment and reward sensitivity stem from the reinforcement sensitivity theory (Gray 1970, 1982, 1987; Gray and McNaughton 2000). According to this theory, people who are sensitive to punishment will have a more negative response to punishment, more attention to punishment-relevant cues and a stronger tendency to avoid punishment. People who are sensitive to reward will have a more positive response to reward, more attention to reward-relevant cues and a stronger tendency to approach reward (Gray 1970; Gray and McNaughton 2000; Davis and Fox 2008).

The attentional system provides the mechanism for detecting and monitoring the environment for stimuli that are relevant to the motivational state of the organism (Mogg and Bradley 1998). People who are heightened punishment sensitive are motivated to avoid punishment and are therefore expected to be more prone to detect punishing signals in the environment; people who are heightened reward sensitive are motivated to obtain reward and are therefore expected to be more prone to detect rewarding signals in the environment (Gray 1970; Gray and McNaughton 2000).

A heightened proneness to detect punishing signals in the environment may result in prolonged anxious states, limited attention for fear-disconfirming information, and feelings of uncontrollability, making people more vulnerable for the development of anxiety disorders (Harvey et al. 2004). A heightened proneness to detect signals of reward may result in positive affect in rewarding situations; however, if the person does not succeed in getting the preferred outcome, this might result in non-reward elicited anger and behavioral problems (Corr 2013) as was found in multiple studies conducted in non-clinical samples of adolescents (Carver 2004; Hundt et al. 2013; Harmon-Jones 2003). Reward and punishment sensitivity are presumed to represent orthogonal dimensions that can vary independently in strength, indicating that all combinations of (relatively) high and (relatively) low PS and RS may be evident in a particular population (Carver and White 1994). Individuals at the far poles of the punishment sensitivity and/or the reward sensitivity dimensions are expected to have an increased risk for developing mental health problems (Pickering and Gray 1999), which might especially become evident during periods with increasing demands, such as adolescence and young adulthood (Leadbeater et al. 2012).

Multiple studies have investigated the associations between self-reported punishment and reward sensitivity and anxiety and behavioral problems, respectively. These self-report measures are well suited to assess the affective component of punishment and reward sensitivity. A review conducted by Bijttebier et al. (2009) indicated that on global measures of mental disorder symptoms, internalizing problems were associated with higher PS, whereas behavioral problems were associated with higher RS. When looking more specifically at anxiety disorders within the internalizing domain, there is ample evidence linking PS to anxiety symptoms in non-clinical child and adult samples (Takahashi et al. 2015; Bijttebier et al. 2009) and linking PS to anxiety disorders in both child and adult clinical samples (Vervoort et al. 2010; Bijttebier et al. 2009). In line with the view that high PS may be a risk factor for the development of anxiety disorders, a longitudinal study showed that (high) self-reported PS in adolescence had predictive value for the level of anxiety symptoms in adulthood, even when controlling for anxiety in adolescence (Izadpanah et al. 2016).

With regard to behavioral disorders, multiple studies indicated an association between RS and behavioral problems. More specifically, RS has been associated with self-reported conduct problems in clinical adolescents (Morgan et al. 2014), trait anger in non-clinical students (Smits and Kuppens 2005; Harmon-Jones 2003), self-reported verbal and physical aggression in non-clinical students (Smits and Kuppens 2005), and self-reported hostility in non-clinical students (Harmon-Jones 2003). Heightened reward sensitivity may result in a higher proneness to detect signals of reward in the environment, higher motivation to approach reward, and may result in positive affect in rewarding situations. However, when more reward sensitive persons experience failures to obtain anticipated reward, this is expected to result in non-reward elicited anger and behavioral problems (Corr 2013; Carver 2004; Hundt et al. 2013; Harmon-Jones 2003).

The evidence mainly stems from cross-sectional studies using self-report measures of punishment and reward sensitivity. However, it is doubtful whether the attention component of punishment and reward sensitivity, namely the proneness to detect cues of punishment and reward respectively, can be adequately assessed by means of self-reports. Performance-based measures seem required to assess this component of RS/PS. Attentional processes help in selecting specific stimuli for further processing and prevent us from being overwhelmed by all information that surrounds us. In this way, it involves the initial filtering of the environment and if there is a bias in this first filtering of information, it might likely contribute to further processing biases that might result in clinical problems (Derryberry and Reed 2002).

In the current study, we therefore decided to use a performance measure (spatial orientation task; Derryberry and Reed 2002) to examine the relation between sensitivity of the punishment system and reward system with anxiety and behavioral problems. The spatial orientation task (SOT) was developed to explore to what extent people direct and hold their attention to places of potential reward and punishment, and was in previous studies successfully used in the context of substance use and addiction in non-clinical adolescent and young adult samples (Colder and O’Connor 2002; van Hemel-Ruiter et al. 2013; van Hemel-Ruiter et al. 2015) eating disorders in clinical and non-clinical adolescents (Jonker et al. 2016; Matton et al. 2017), and depression in clinical adolescents and young adults (Vrijen et al. 2018).

The SOT is a reaction time task which consists of games in which participants can gain points (winning games), and games where points can be lost (losing games). Before each target appears, a cue is presented that either signals a high chance of reward (in winning games)/non-punishment (in losing games) or a high chance of punishment (in losing games)/non-reward (in winning games). The target can occur either in the cued or uncued location. The difference in reaction time between the cued and uncued location represents the cue validity effect. This cue validity effect indicates the attentional bias of individuals to cues predicting punishment or reward. Separate cue validity scores were calculated for short (250 ms) and long (500 ms) delays between cues and targets, which provides the opportunity to examine the relative importance of early (short delay) attentional processes and attentional processes that allow for some regulatory control (long delay) (Derryberry and Reed 2002).

Previous cross-sectional research indicated that anxious students showed an enhanced cue validity effect for cues signaling punishment (Derryberry and Reed 2002). This effect was found with short cue delays, but not with longer cue delays (Derryberry and Reed 1994, 2002) and is suggested to be largely automatic (McNally 1995; Mogg et al. 1995). Allocating attention to objectively threatening stimuli can be regarded as an adaptive mechanism that serves rapid detection and avoidance of danger (Mogg and Bradley 1998). However, an attentional bias to subjective or ambiguous threat may contribute to the development and maintenance of anxiety problems.

An enhanced cue validity effect for non-punishing cues may reflect a tendency to seek safety. Within a threatening situation, attention to safety may help the person attenuate their anxiety, enable to remain in, and learn from, the environment. This may generally be adaptive, however, an enhanced cue validity effect for non-punishing cues may prevent the habituation and reappraisal of stimuli perceived to be threatening, and thereby maintain anxiety (Harvey et al. 2004; MacLeod and Grafton 2016). Previous research indeed found evidence indicating that high anxious individuals showed an heightened cue validity effect for cues signaling non-punishment (regarded as safety cues (Derryberry and Reed 2002). This effect was evident for cues with longer cue delay, suggesting that this process may be less automatic and more voluntary.

Increased reward sensitivity leading to enhanced responses to reward is assumed in young children with clinical behavioral problems (Quay 1993). Previous research showed that enhanced attentional engagement to cues signaling reward and difficulty disengaging from cues signaling reward were related to adolescent substance use (van Hemel-Ruiter et al. 2013; Colder and O’Connor 2002), and that this bias measured during adolescence was predictive for substance use in young adulthood (van Hemel-Ruiter et al. 2015). Furthermore, it was found that an attentional bias to reward, measured with an adapted version of the Posner spatial attention-cueing task, was associated with behavioral problems in 5 year old children (He et al. 2016). It is however untested whether an attentional bias to reward as indexed by a spatial orientation task is related to and has prognostic value for the development of behavioral problems in adolescence and young adulthood.

The current study was designed to investigate how individual differences in attentional bias for cues predicting punishment and reward are associated with symptoms of anxiety and behavioral disorders in adolescence and young adulthood.

We will try to replicate the findings from Derryberry and Reed (2002) in an adolescent sample to see whether (i) having a stronger cue validity effect for cues signaling punishment with short cue delay is associated with higher anxiety symptoms. We will extend previous research by also looking at (ii) the prognostic value of this cue validity effect for cues signaling punishment with short cue delay for anxiety symptoms at six years follow-up. We (iii) will also try to replicate the findings from Derryberry and Reed (2002) in an adolescent sample to see whether a stronger cue validity effect for cues signaling non-punishment with long cue delay is associated with having higher anxiety symptoms and extend this line of research by (iv) also looking at the prognostic value of this cue validity effect at six years follow-up. Our study is the first study to investigate an attentional bias for reward on behavioral problems in adolescence and young adulthood. Based on studies investigating the role of an attentional bias for reward on substance use in adolescents and young adults (van Hemel-Ruiter et al. 2013; van Hemel-Ruiter et al. 2015; Colder and O’Connor 2002) and a reward bias on behavioral problems in young children (He et al. 2016) we will (v) test whether a stronger cue validity effect for cues signaling reward with both short and long cue delay is associated with behavioral problems in adolescents and has (vi) prognostic value for behavioral problems at 6 years follow-up. We expect (vii) this association between a stronger cue validity effect for cues signaling reward with behavioral problems to be most pronounced on trials with long cue delay since than both automatic and more voluntary processes are expected to play a role and are expected to have an added effect.

## Method

This study is preregistered on Open Science Framework, the preregistration can be found via https://osf.io/pbw6h.

### Participants

Participants of the Tracking Adolescent’s Individual Lives Survey (TRAILS) were included in this study. TRAILS is a large prospective population study of Dutch adolescents coming from five northern municipalities in the Netherlands including both rural and urban areas. Children born between 1 October 1989 and 30 September 1990 from two northern municipalities and children born between 1 October 1990 and 30 September 1991 from the remaining three northern municipalities form the TRAILS cohort. At baseline (T1), 2230 children were included, with assessments taking place in 2001 and 2002 (Huisman et al. 2008; Ormel et al. 2012). Written informed consent was obtained from all adolescents and their parents.

The current study reports on data from the third (T3) and fifth (T5) assessment waves, data collection of T3 took place between 2005 and 2007, 1816 adolescents participated (81,4% of the initial sample) with a mean age of 16.3. The fifth wave (T5) was conducted in 2012 and 2013; 1778 adolescents participated (80% of the initial sample). Participants were then between 21 and 24 years of age, with a mean age of 22.3 years (Kretschmer et al. 2015). Anxiety and behavioral problems were assessed during the T3 and T5 assessments.

The SOT was the first task in a series of laboratory tasks that were performed in addition to the general assessments during T3. For these laboratory tasks a focus group of 744 participants was contacted, 715 (96%) of these agreed to participate. This focus group is overrepresented by adolescents with a high risk of mental health problems. High risk was based on temperament (high frustration and fearfulness, low effortful control), lifetime parental psychopathology (depression, anxiety, addiction, antisocial behavior or psychoses) and/or living in a single parent family. Of this focus group, 66.2% had at least one of these risk factors. The remaining 33.8% were randomly selected from the low-risk TRAILS participants (for more information see supplement Table S1, see also Jonker et al. 2016).

**Fig. 1:**
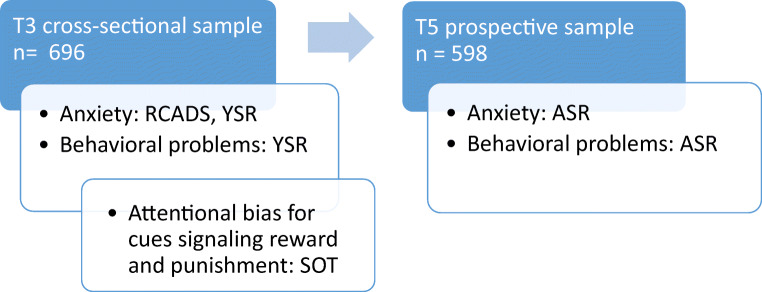
Overview of the timeline, sample size and measurements of the study

For the cross-sectional part of the study, participants who participated in the behavior measure of reward and punishment sensitivity (SOT, T3) and who also completed the questionnaires measuring anxiety and behavioral problems on T3 were selected using listwise deletion. For the prospective part of the study, participants who participated in the behavior measure of reward and punishment sensitivity at T3, and who also completed the questionnaires measuring anxiety and behavioral problems on T5 were selected using listwise deletion. Figure 1 gives an overview of the timeline, sample size, and measurements of the study. Table 1 gives an overview of the characteristics of the samples.

**Table 1:** Sample characteristics

	*M (SD)* or percentage	
	T3	T5
Cross-sectional sample (*N* = 696)		
Age	16.14 (0.60)	–
Gender % female	51	–
*Anxiety*		–
RCADS mean item score	1.69 (1.21)	
YSR mean item score	0.30 (0.30)	
*Behavioral problems*		–
YSR mean item score	0.31 (0.24)	
Prospective sample (*N* = 598)		
Age	16.15 (0.59)	22.20 (0.63)
Gender % female	53	53
*Anxiety*		
RCADS mean item score	1.70 (1.21)	
YSR/ASR mean item score	0.31 (0.30)	0.40 (0.37)
*Behavioral problems*		
YSR ASR mean item score	0.31 (0.24)	0.22 (0.24)

*RCADS* Revised Child Anxiety and Depression Scale-Child version, *YSR* Youth Self Report, *ASR* Adult Self Report

### Measures

#### Anxiety

At T3 anxiety was assessed using the Revised Child Anxiety and Depression Scale-Child version (RCADS-C). The RCADS-C (Chorpita et al. 2000; Dutch version: Muris et al. 2002) consists of 47 items that measures symptoms of DSM-IV anxiety disorders and depression in children from the ages of 7 to 19. The RCADS-C has six subscales; separation anxiety disorder, social phobia, generalized anxiety disorder, obsessive compulsive disorder, panic disorder, and major depressive disorder. In the current study, we used a total score consisting of only those subscales that correspond to the primary anxiety disorders of children; separation anxiety disorder (7 items, Cronbach’s *α* = 0.63), social phobia (9 items, Cronbach’s *α* = 0.86), generalized anxiety disorder (6 items, Cronbach’s *α* = 0.79), and panic disorder (9 items, Cronbach’s *α* = 0.77), which is also in line with the DSM 5 categorization of anxiety disorders. The RCADS was found to have good internal consistency (Chorpita et al. 2005; Donnely et al. 2019), good 1 week test-retest reliability (Chorpita et al. 2005), good convergent validity (Esbjørn et al. 2012; Bouvard et al. 2015; Donnely et al. 2019) and concurrent validity (Chorpita et al. 2005)

At T3, anxiety was also assessed with the Youth Self Report (YSR), which consists of 112 items on behavioral and emotional problems in the past 6 months (Achenbach et al. 2003). Internal consistency of the total scale is 0.96 and ranges from 0.60 to 0.90 for the subscales. The test-retest reliability for the total scale is 0.87 and ranges from 0.67 to 0.91 for the subscales. Construct- and criterium-related validity were found to be adequate (Verhulst et al. 1997).

The DSM-IV Anxiety problems subscale of the YSR (6 items, Cronbach’s *α* = 0.65) was included in the current study. At T5, anxiety was assessed using the adult version of the Adult Self Report (ASR) which consist of 102 items on behavioral and emotion problems in the past 6 months (Achtenbach and Rescorla 2001; Achenbach et al. 2003). The reliability and validity of the ASR were found to be good (Achenbach et al. 2003). The DSM-IV anxiety problems subscale of the ASR (7 items, Cronbach’s *α* = 0.76) was included in the current study.

#### Behavioral Problems

At T3, behavioral problems were assessed with the Youth Self Report (YSR) (Achtenbach and Rescorla 2001; Achenbach et al. 2003), using the aggressive behavior subscale (17 items, Cronbach’s *α* = 0.81). At T5 behavioral problems were assessed with the adult version of the Youth Self Report, namely the Adult Self Report (ASR), using the aggressive behavior subscale (15 items, Cronbach’s *α* = 0.84).

#### Spatial Orientation Task (SOT)

The SOT was developed to examine individuals’ inclination to direct and hold their attention to cues signaling reward and punishment (Derryberry and Reed 2002). Participants have to respond as quickly as possible to a neutral target that is preceded by a cue in order to gain points or to avoid losing points. They have to press the ‘b’ key as soon as they see the target. Their score is displayed in the middle of the screen. There are two types of games, in losing games, participant lose 10 points if they respond too slowly, and their score remains unchanged if they respond sufficiently fast, whereas in winning games, participant win 10 points if they respond sufficiently fast, and their score remains unchanged if they respond sufficiently fast. At the beginning of the task, participants were told that those with the highest scores in the winning games would win an attractive prize (e.g. a balloon ride) and that an extremely low score on the losing games would result in having to redo the task until their performance was good enough. Participants lose 10 points regardless of the game type when they respond when no target appears (catch trials) or before the target has appeared. The task consists of four losing and four winning games, which are alternated every two games. Each game consists of 32 cued, 16 uncued and 8 catch trials that are presented randomly. Before these eight games, participants get four training games (two losing and two winning) each consisting of 6 cued, 6 uncued and 2 catch trials. The task was performed on an Intel Pentium 4 CPU computer with a Philips Brilliance 190 P monitor and run by E prime software version 1.1. (Psychology Software Tools Inc., Pittsburgh, Pennsylvania). Participants were seated 50 cm away from the screen and responses were collected on the computers’ keyboard (Jonker et al. 2016). Fig. S1 under supplements shows the SOT task, which can be helpful in addition to the text description of the task.

### Components of the SOT Task

#### Cued or Uncued

Each trial starts with the appearance of two vertical black bars on a white background, left and right of the participant’s score that is presented in the middle of the screen. This score was set to zero at the beginning of each block. A new trial is signaled by the current score disappearing from the screen for 200 ms after which it reappeared. After a 250 ms delay, a cue replaced one of the two black bars. Then after a delay of either 250 (short delay) or 500 ms (long delay) the target appeared either centered within the cue or centered within the remaining black bar on the other side of the screen. When the target appears in the cue, the trial is called a cued trial, when the target appears in the uncued black bar, the trial is called an uncued trial. This cue operates as a signal of reward/non-punishment or punishment/non-reward by indicating the change of winning or losing points.

#### Signals of Reward/Non-punishment and Punishment/Non-reward

The task included two different cues that could precede the target; a blue arrow pointing upwards and a red arrow pointing downwards. Participants were informed that both cues indicated the probable location of the target, with 2/3 of the targets appearing in the cued location. It was explained that in general the blue cue was a signal for having a high change of responding fast enough (fast enough 75% of the time when cued, 25% of the time when uncued), whereas the red cue was a signal for having a high change of a too slow response (fast enough 25% of the time when cued, 75% of the time fast enough when uncued). So in general the blue arrow becomes a signal of reward (in winning games) or non-punishment (in losing games) because the chance of being fast enough is high, and the red arrow becomes a signal of non-reward (in winning games) or punishment (in losing games) because the chance of not being fast enough is high. Lastly, participants were informed that there occasionally would be trials where no target appeared.

#### Feedback

After 500 ms in each response (or 1 s in the case of catch trial), the cue and target are removed, and the two black bars appear again. A feedback signal is given below the score. Both the blue upward arrow and the red downward arrow were also used as a feedback signal. The blue arrow signaled a fast enough response on targeted trials or a correct nonresponse on catch trials. The red arrow signaled a too slow response on targeted trials or an inappropriate response on catch trials. After 250 ms the score is updated if necessary.

#### Calculation of Cutoffs for Fast and Slow Responses

At the end of each game the participant’s median reaction time and standard deviation based on all trials in that game were calculated to compute cutoffs for fast and slow responses in the following game of the same type. For the first two practice blocks a fixed cutoff of 350 ms was used since no personalized cutoffs were available for these blocks. During easy trials (cued blue or uncued red) responses were labeled sufficiently fast when they were faster than participant’s median reaction time plus 0.55 times the standard deviation. During hard trials (uncued blue or cued red) responses were labeled sufficiently fast when they were faster than participants’ median reaction time minus 0.55 times the standard deviation. Further, since reaction times tend to be about 25 ms slower after a short cue delay time than after a long cue delay time, 12 ms were added to the median reaction time for short-delay trials and 12 ms were subtracted from the median reaction time for long-delay trials (Derryberry and Reed 2002). This was done after the median reaction time for that game was calculated.

### Procedure

This study reports on data of a large prospective cohort study; in the current study a cross sectional as well as prospective approach were taken. The Dutch (national) Central Committee on Research Involving Human Subjects (CCMO) approved the study. Participants provided informed consents. Anxiety and behavioral problems were measured with self-reports during the regular assessments at T3 and T5, which took place at the TRAILS offices. The laboratory tests including the SOT were assessed at selected locations in the town of residence of participants, in a sound-attenuating room with blinded windows. In order to optimize standardization of the experimental session, test-assistants received extensive training.

### Calculation of Indices of Punishment and Reward Sensitivity

Given the design of the task, the blue cue signals reward (in winning games) or non-punishment (in losing games), and the red cue signals non-reward (in winning games) or punishment (in losing games). In general, it is expected that people have a preference to direct attention to cues that signal reward or non-punishment compared to cues that predict punishment and non-reward. Furthermore, it is expected that participants in general have more difficulty to disengage from cues signaling reward/non-punishment than from cues signaling non-reward/punishment. In line with previous studies (Jonker et al. 2016; van Hemel-Ruiter et al. 2015) we carried out a series of paired sample *t*-tests to test whether the SOT worked as expected. More specifically, we compared the mean reaction times in cued blue versus cued red trials as well as in uncued blue versus uncued red trials. We expected faster responses in cued blue trials compared to cued read trials, and faster responses on uncued red compared to uncued blue trials.

In line with He et al. (2016), the proneness to attend to rewarding/non-punishing cues was indexed by the cue validity effect for cues signaling reward/non-punishment. The mean reaction time to cued blue trials (signaling high chance of reward in winning games/non-punishment in losing games) was subtracted from the mean reaction time to uncued blue trials, where in general people are expected to be slower on uncued trials, leading to a positive difference score. It is expected that this difference is larger for people who are more prone to attend to rewarding/non-punishing cues and therefore are more slow when the target appears in the uncued condition compared to the cued condition when the cues signals a high chance of reward/non-punishment.

Similarly, the cue validity effect for cues signaling punishment/non-reward was computed by subtracting the mean reaction time to cued red trials (signaling a high chance of punishment in losing games/non-reward in winning games) from the mean reaction time to uncued red trials, where in general people are expected to be slower on uncued trials, leading to a positive difference score. It is expected that this difference is larger for people who are more prone to attend to punishing/non-rewarding cues and therefore are more slow when the target appears in the uncued condition compared to the cued condition when the cue signals a high chance of punishment/non-reward. In order to take individual differences in reaction times into account when calculating the cue validity effects, we subtracted the individual’s mean reaction time on the practice trials on either cued or uncued trials from the corresponding mean scores. Subtracting these means was not mentioned in the preregistration but was included after ample discussions about ways to improve the reliability of the task. This subtraction reduces the correlation between the components (RT of cued trials and RT of uncued trials) of the cue validity effects and thereby should improve the reliability of the AB-measures. See Table 2 for the calculations of the cue validity effects and the estimates of the reliability controlled and uncontrolled for the individual’s mean reaction time for cued and uncued trials. As can be seen, the reliability of the measure indeed improved after subtracting the individual’s mean reaction time on practice trials. The estimates of the reliability of the controlled cue validity effects indicate that each of the calculated cue-validity effects has adequate reliability. The results of the analyses that strictly followed the preregistration can be found in Appendix B.

**Table 2:** Calculation of the cue validity effects controlled for mean reaction time, the interpretation of the cue validity effects, and reliability estimates of the controlled and uncontrolled cue validity effects

Reward and Punishment indices	Game	Calculation	Interpretation	Cue delay time	Reliability estimate Spearman-Brown coefficient controlled for individuals’ mean reaction time (uncontrolled)
Cue validity effect for cues signaling reward	Winning game	(mean RT uncued blue trials – mean RT uncued practice trials) – (mean RT cued blue trials – mean RT cued practice trials)	High score: stronger cue validity effect for cues signaling reward	250 ms	0.795 (0.527)
				500 ms	0.728 (0.430)
Cue validity effect for cues signaling non-reward		(mean RT uncued red trials – mean RT uncued practice trials) – (mean RT cued red trials – mean RT cued practice trials)	High score: stronger cue validity effect for cues signaling non-reward	250 ms	0.765 (0.527)
				500 ms	0.725 (0.350)
Cue validity effect for cues signaling punishment	Losing game	(mean RT uncued red trials – mean RT uncued practice trials) – (mean RT cued red trials – mean RT cued practice trials)	High score: stronger cue validity effect for cues signaling punishment	250 ms	0.800 (0.541)
				500 ms	0.745 (0.385)
Cue validity effect for cues signaling non-punishment		(mean RT uncued blue trials – mean RT uncued practice trials) – (mean RT cued blue trials – mean RT cued practice trials)	High score: stronger cue validity effect for cues signaling non-punishment.	250 ms	0.793 (0.499)
				500 ms	0.689 (0.262)

*RT* Reaction time

### Statistical Analyses

As step 1, bivariate correlations were calculated between the cue validity effects, anxiety symptoms, and behavioral problems to examine the bivariate relationships between the variables. Step 2 consisted of the main analyses, where we performed multiple regression analyses. The cue validity effects from the losing games were used as predictors for anxiety symptoms, whereas the cue validity effects from the winning games were used as predictor variables for behavioral problems. We conducted both cross-sectional analyses using T3 anxiety symptoms and behavioral problems as dependent variables, and prospective analyses using T5 anxiety symptoms and behavioral problems as dependent variables.

Step 3 consisted of exploratory analyses where we tested whether effects are game specific by conducting the regression analyses with the cue validity effects from the winning games for anxiety symptoms and the cue validity effects from the losing games for behavioral problems, both cross-sectionally and prospectively. Furthermore, we tested whether the cue validity effects predicted change in anxiety and behavioral problems by conducting a hierarchical regression analysis with the cue validity effects from the losing games on anxiety symptoms at T5, when statistically controlling for anxiety symptoms at T3. Similarly, we conducted a hierarchical regression analysis with the cue validity effects of the winning games on behavioral problems at T5, when statistically controlling for behavioral problems at T3.

Step 4, if we would find that both the cue validity effects of the losing and winning games predicted scores in either behavioral problems and/or anxiety we would perform a regression analysis on that outcome variable including all eight cue validity effects (from both the winning and losing games) to see whether they explain the same variance or have (also) unique contributions.

### Alpha Correction

Since we test our main hypothesis on anxiety symptoms with two separate regression analyses (cross-sectional and prospective) and our main hypothesis on behavioral problems with two separate regressions, all including four independent variables, we corrected for multiple testing. The Bonferroni-Holm correction for multiple comparisons was used (Holm 1979), with 0.0125, 0.0250, 0.0375 and 0.050 as alpha levels.

## Results

We have missing data in the current study, which can produce biased estimates due to differences between missing and included participants and can reduce the statistical power of a study, leading to invalid conclusions (Kang 2013). Therefore, we checked whether the missing data in our study would pose a threat to the validity of our conclusions due to having a biased sample or a too large reduction in power.

For the cross-sectional sample, we could include 696 of the 715 participants (97%). This means that for the cross-sectional analyses only 3% is missing, indicating that bias and loss of power are both likely to be inconsequential (Graham 2009). Furthermore, for the prospective sample, including participants with complete data only, did not seem to lead to a biased sample, given that we did not find any significant differences between the individuals with missing prospective data (*n* = 117, 16%) and individuals with complete prospective data (*n* = 598) on anxiety and behavioral problems at t3 as well as with regard to the cue validity effects.^1^ With regard to power, a sample size of 544 participants is needed to be able to find an effect with a small effect size, α of 0.0125 and power of 0.80. Therefore, given our sample size of 598 participants, power should also not be a problem for the prospective analyses.

### Descriptives

In line with Jonker et al. (2016), trials during which participants did not respond to the target were deleted, which resulted in deletion of 3.3% of the trials. Also trials on which participants responded before the target appeared were removed, resulting in the deletion of 8.3% of the trials. Furthermore, reaction times below 125 ms, which are expected to be anticipation errors, were deleted, resulting in the deletion of 8.5% of the remaining trials. The mean reaction times for each game type (winning and losing) and trial type (easy cue/hard cue and cued/uncued) were calculated after these deletions and are presented in Tables 3 and 4.

**Table 3:** Mean reaction times and standard deviations of the Spatial Orientation Task in the cross-sectional sample

	Cued		Uncued	
	Blue	Red	Blue	red
Losing game				
Short cue delay time (250 ms)	330 (47)	358 (53)	456 (89)	458 (92)
Long cue delay time (500 ms)	332 (59)	366 (68)	380 (83)	374 (79)
Winning game				
Short cue delay time (250 ms)	336 (43)	366 (48)	468 (90)	471 (90)
Long cue delay time (500 ms)	342 (58)	379 (67)	384 (79)	377 (74)

*n* = 696

**Table 4:** Mean reaction times and standard deviations of the Spatial Oriental Task in the prospective sample

	Cued		Uncued	
	Blue	Red	Blue	red
Losing game				
Short cue delay time (250 ms)	327 (43)	356 (49)	454 (86)	458 (92)
Long cue delay time (500 ms)	330 (56)	363 (66)	378 (80)	373 (75)
Winning game				
Short cue delay time (250 ms)	335 (41)	364 (46)	466 (89)	468 (88)
Long cue delay time (500 ms)	341 (57)	377 (67)	380 (77)	375 (72)

*n* = 598

### Task Design Check

In line with previous studies (Jonker et al. 2016; van Hemel-Ruiter et al. 2015) a series of paired sample *t*-tests were carried out to test the expectation that people in general respond faster to cued blue trials compared to cued red trials, and have faster responses on uncued red compared to uncued blue trials (see Table 5). Participants were faster on the cued blue than cued red trials for both winning and losing games, irrespective of the cue delay time, indicating a general preference to direct attention to cues that predict reward or non-punishment compared to cues that predict punishment and non-reward. Thus, in line with the task design, participants showed a generally enhanced attentional engagement to stimuli signaling reward and non-punishment. Furthermore, participants were slower on uncued blue trials than uncued red trials on long cue delay time trials in winning games, indicating a difficulty to disengage from reward with longer cue delay.

**Table 5:** Task design check; differences between red and blue targets

	Calculation	95% CI for the difference		*p*
		Lower bound	Upper bound	
Short cue delay time (250 ms)				
Wining game	Cued red – cued blue	28.07	32.85	<0.001*
	Uncued red- uncued blue	−2.24	7.03	0.311
Losing game	Cued red- cued blue	25.34	30.62	<0.001*
	Uncued red – uncued blue	−2.42	7.23	0.328
Long cue delay time (500 ms)				
Winning game	Cued red-cued blue	32.63	39.94	<0.001*
	Uncued red- uncued blue	−11.35	−3.00	0.001*
Losing game	Cued red – cued blue	29.87	37.67	<0.001*
	Uncued red – uncued blue	−10.35	−1.33	0.011*

*n* = 696 * *p* < 0.05, *CI* confidence interval

### Step 1

Bivariate correlations were calculated between the cue validity effects, anxiety symptoms, and behavioral problems, see Table 6.

**Table 6:** Bivariate correlations of cue validity effects with internalizing and behavioral problems at T3 and T5

	1	2	3	4	5	6	7	8	9	10	11	12	13
1 Anxiety 3 (RCADS)	–												
2 Anxiety t3 (YSR)	0.713*	–											
3 Behavioral problems t3	0.288*	0.271*	–										
4 Anxiety t5	0.467*	0.473*	0.224*	–									
5 Behavioral problems t5	0.385*	0.337*	0.388*	0.641*	–								
6 CV-reward short	−0.061	−0.065	−0.065	−0.056	0.058	–							
7 CV-reward long	−0.067	−0.081	−0.046	−0.057	−0.046	0.702*	–						
8 CV-nonreward short	−0.044	−0.037	−0.048	−0.043	−0.040	0.897*	0.706*	–					
9 CV-nonreward long	−0.072	−0.091	−0.056	−0.070	−0.065	0.654*	0.748*	0.689*	–				
10 CV-punishment short	−0.018	0.030	−0.055	−0.009	−0.007	0.803*	0.710*	0.807*	0.672*	–			
11 CV-punishment long	−0.094	−0.087	−0.022	−0.087	−0.062	0.646*	0.717*	0.671*	0.741*	0.692*	–		
12 CV-nonpunishment short	−0.067	−0.065	−0.034	0.067	−0.068	0.795*	0.676*	0.769*	0.665*	0.795*	0.669*	–	
13 CV-nonpunishment long	−0.070	−0.066	−0.034	−0.095	−0.084	0.698*	0.741*	0.681*	0.723*	0.697*	0.746*	0.697*	–

Correlations between T3 variables and CV variables are based on a sample size of *n* = 696, correlations with T5 variables are based on a sample size of *n* = 598. * *p* < 0.01

Significant correlations were found between anxiety at T3 and T5 and behavioral problems at T3 and T5. Furthermore, significant correlations were found between anxiety and behavioral problems. However, no significant bivariate relationships were found between the cue validity effects and anxiety or behavioral problems.

### Step 2 Main Analyses

Unexpectedly, no significant bivariate relationships were found between the cue validity effects and anxiety or behavioral problems. The planned main analyses were still conducted to test whether the whole model including multiple cue validity effects was significant, which would be indicative of their joint effects.

#### Cross-Sectional Analyses

Anxiety symptoms (T3): No significant associations between the cue validity effects for cues signaling punishment or non-punishment with anxiety were found (see Table 7). The full model was also not significant.

**Table 7:** regression model with anxiety (T3) and cue validity effects for punishment and non-punishment

Dependent variable Anxiety T3	*b*	*SE b*	*Beta*	*t*	*p*
Constant b0	.414	0.019		21.50	<0.001
CV-punishment-short	0.000	0.000	0.158	2.34	0.019
CV-punishment-long	0.000	0.000	−0.124	−2.02	0.044
CV-non-punishment-short	0.000	0.000	−0.094	−1.41	0.159
CV-non-punishment-long	0.000	0.000	−0.023	−0.373	0.709
*R*^*2*^_*change*_. *=* 0.017	*F* = 2.93	*p =* 0.020			

*n* = 696

Behavioral problems (T3): No significant associations between the cue validity effects for cues signaling reward or non-reward with behavioral problems were found (see Table 8).

**Table 8:** Regression model with behavioral problems (T3) and cue validity effects for reward and non-reward

Dependent variable Behavioral problems T3	*b*	*SE b*	*Beta*	*t*	*p*
Constant b0	0.318	0.017		18.51	<0.001
CV-reward-short	0.000	0.000	−0.070	−1.05	0.295
CV-reward-long	0.000	0.000	0.014	0.23	0.822
CV-non-reward-short	0.000	0.000	0.024	0.35	0.729
CV-non-reward-long	0.000	0.000	−0.038	−0.62	0.537
*R*^*2*^_*change*_. *=* 0.005	*F* = 0.85	*p =* 0.494			

*n* = 696

#### Prospective Analyses

Anxiety symptoms (T5): It was found that having a larger attentional bias to cues signaling punishment with short cue delay predicted higher anxiety symptoms. The full model was significant (see Table 9).

**Table 9:** Regression model with anxiety (T5) and cue validity effects for punishment and non-punishment

Dependent variable Anxiety T5	*b*	*SE b*	*Beta*	*t*	*p*
Constant b0	0.380	0.026		14.74	<0.001
CV-punishment-short	0.001	0.000	0.192	2.64	0.008*
CV-punishment-long	0.000	0.000	−0.080	−1.22	0.222
CV-non-punishment-short	0.000	0.000	−0.090	−1.24	0.216
CV-non-punishment-long	0.000	0.000	−0.107	−1.56	0.120
*R*^*2*^_*change*_. *=* 0.021	*F* = 3.22	*p =* 0.012*			

*n* = 598,* significant at *p* < 0.0125

Behavioral problems: The cue validity effects for cues signaling reward or non-reward did not predict behavioral problems (see Table 10).

**Table 10:** Regression model with behavioral problems (T5) and cue validity effects for reward and non-reward

Dependent variable Behavioral problems T5	*b*	*SE b*	*Beta*	*t*	*p*
Constant b0	0.216	0.018		11.88	<0.001
CV-reward-short	0.000	0.000	−0.062	−0.86	0.393
CV-reward-long	0.000	0.000	0.020	0.29	0.776
CV-non-reward-short	0.000	0.000	0.044	0.59	0.555
CV-non-reward-long	0.000	0.000	−0.069	−1.04	0.298
*R*^*2*^_*change*_. *=* 0.005	*F* = 0.82	*p =* 0.514			

*n* = 598

### Step 3 Exploratory Analyses

In order to see whether effects are game specific, we conducted the same analyses also with the cue validity effects with the other game type. We did not find significant associations with these analyses. Furthermore, we investigated whether the cue validity effects predicted change in anxiety and behavioral disorder symptoms from T3 to T5. We did not find significant effects. The findings of these exploratory analyses can be found in Appendix A.

### Step 4 (Only when Necessary Based on Previous Results)

We did not find effects in both the losing and winning games on either behavioral problems or anxiety symptoms. Therefore, this step was not conducted.

## Discussion

The major findings can be summarized as follows: first, the cue validity effect for cues signaling punishment with short cue delay showed no positive bivariate association with concurrent or prospective anxiety symptoms. Second, cue validity effects of cues signaling non-punishment showed no concurrent or prospective bivariate associations with anxiety. Third, independent of cue delay, the cue validity effect for cues signaling reward showed no positive concurrent or prospective bivariate association with behavioral problems. Only one of the expected associations was found to be significant in the regression analyses. In this regression analysis, it was found that the cue validity effect for cues signaling punishment with short cue delay showed a prognostic relationship with anxiety symptoms at 6 years follow-up. However, the failure to find a similar relationship in the bivariate analysis clearly questions the robustness of the prognostic value of the attentional bias for cues predicting punishment. Given that we looked in two large samples selected from a large representative cohort sample, we should have been able to find the predicted associations if they would exist. Overall, our study does not seem to support the role of attentional proneness to general cues of punishment or reward as risk factors for the development of anxiety and behavioral problems respectively.

### Attentional Bias for Cues Signaling Punishment and Anxiety Symptoms

Unexpectantly, our study does not seem to support the role of an attentional bias for cues signaling punishment as risk factor for the development of anxiety symptoms. It seems that the attentional proneness component for general cues of punishment is not the relevant component of punishment sensitivity to focus on in predicting anxiety symptoms in young adulthood. According to the cognitive-motivational view (Mogg and Bradley 1998) attentional biases may not necessarily play a major causal role in the etiology of clinical anxiety states. They indicate that attentional biases are also likely to be found in low anxious individuals when external stimuli have high threat value. This cognitive-motivational view does not exclude the possibility that attentional biases are important in the maintenance of clinical anxiety especially when automatic orienting to threat may be accompanied by avoidant action tendencies. This is likely to result in increased detection of minor threat in the environment without prolonged exposure. As a result, the threat expectation is not falsified and anxiety is maintained in the long term. This is in line with a meta-analysis indicating that children with anxiety showed a significantly greater bias to threat-related stimuli compared to controls (Dudeney et al. 2015), which, combined with our findings, would indicate that this bias might not be so much a risk factor but more a symptom or maintaining factor. Recent findings on positive outcomes from attentional bias trainings away from threat (Price et al. 2016) suggest that it might indeed play a causal role in the maintenance of anxiety. Therefore, we recommend future research to investigate attentional biases as possible maintaining factor of anxiety.

### Attentional Bias for Non-punishing Cues and Anxiety Symptoms

We hypothesized that people with higher anxiety symptoms would also show an attentional bias for cues of non-punishment (safety) as was found in a previous study by Derryberry and Reed (2002). However, we did not find this effect. According to Gray and McNaughton (2000), the reward sensitivity system is sensitive to signals of reward, non-punishment and escape from punishment, whereas the punishment sensitivity system is sensitive to signals of punishment, non-reward and novelty. Therefore, Gray’s theory would predict that it is the reward and not the punishment sensitivity system that is responsive to cues signaling non-punishment, and therefore would not predict an attentional proneness to cues of non-punishment but only to punishing cues in anxious people. Our results are in line with this expectation based on Gray’s theory. Other studies indicating that anxious people attend to safety cues were conducted with more specific threat stimuli, such as images reflecting mild injuries within the context of blood-injection-injury fears (e.g., Mogg and Bradley 2004). It might be that people with anxiety disorders attend to safety mainly in situations where they are confronted with highly threatening stimuli that are specific to their anxiety problems. These findings seem also more indicative of attentional proneness to be a possible maintaining factor rather than a risk factor for developing anxiety problems.

### Attentional Bias for Cues Signaling Reward and Behavioral Problems

Unexpectedly, we did not find an association between attentional bias for rewarding cues and behavioral problems, neither cross-sectionally nor prospectively. Previous studies did find an association between a stronger cue validity effect for rewarding cues and behavioral problems (He et al. 2016; Morales et al. 2015), however these studies were conducted in young children, aged 3–5 and the behavioral problem scale used in this study measures both behavioral problems and ADHD symptoms. We therefore do not know whether the association was found because of the behavioral problems and/or ADHD symptoms. Furthermore, they did not use the SOT to index the cue validity effect, which might also explain differences in findings. It might also be that later in life more variability in factors contributing to behavioral problems come into play that either are more important in their contribution to behavioral problems or that moderate the association between proneness to rewarding cues and behavioral problems. Some studies indicate that pubertal development is associated with higher reward sensitivity (Urosevic et al. 2014). At age 16 there might be some variability in pubertal development which might interfere with our measure of reward sensitivity and might therefore attenuate the association between reward sensitivity and behavioral problems.

Studies using the same SOT task in the same sample found that decreased reward responsiveness, as indicated by difficulties in shifting attention from expected non-reward to expected reward at age 16 predicted depression during follow-up. Adolescents that have more difficulty to shift attention from negative to potentially rewarding situations might process disproportionally more negative information, making them more vulnerable for depression (Vrijen et al. 2018). Furthermore, facilitated attention towards rewarding cues was associated with substance abuse in adolescents (van Hemel-Ruiter et al. 2013). This facilitated attention toward appetitive cues may lead to a more detailed and sustained processing of the positive effects of substance use, and may therefore increase the likelihood that the association between these cues and positive effects will be stored in memory, increasing arousal and attentional bias for these positive cues and it may lower the threshold for eliciting craving. Apparently, the association between reward sensitivity and behavioral problems is less clear. We expected that increased reward sensitivity would contribute to behavioral problems because it would make people more vulnerable to experience negative affect and problematic behaviors when they experience non-reward situations (Corr 2013; Carver 2004; Hundt et al. 2013; Harmon-Jones 2003). However, in this study we do not know whether people indeed regularly experienced these non-reward situations. It might be that there are more vulnerable groups, such as adolescent from ethnic minorities, lower social economic status, coming from a divorced family or following special education, that might more frequently encounter situations involving non-reward, which might than foster the association between reward sensitivity and behavioral problems. We encourage future research to look at these vulnerable groups.

Reward sensitivity can give rise to either a positive or negative emotional stage, such as hope or frustration/anger. For non-clinical children reward sensitivity may not be associated with behavioral problems but rather with positive social and environmental functioning when children are able to adjust their reward seeking behavior to possible risks (Rawal et al. 2013). Therefore, given our findings, it seems that attentional proneness to rewarding cues is not a clear risk factor for the development of behavioral problems in young adulthood.

## Conclusion

Our study is the first prospective study investigating attentional proneness for punishing cues as risk factor for anxiety, and attentional proneness for rewarding cues as risk factor for behavioral problems in young adulthood. Our findings do not seem to support the role of these attentional biases as risk factors for the development of anxiety and behavioral problems.

## Electronic supplementary material

ESM 1 (DOCX 83.1 kb)

ESM 2 (DOCX 20 kb)

ESM 3 (DOCX 46.6 kb)

## Footnotes

1 We also conducted the prospective regression analysis on anxiety symptoms predicted by the cue validity effects to punishing and non-punishing cues using a simple multiple imputation model. Gender, anxiety and behavioral problem variables from t3 and t5 (both self-report and parent report, namely; RCADS-t3, YSR-anxiety-t3, CBCL-anxiety-t3, ABCL-anxiety-t5, BAI-t5, YSR-aggressive-t3, CBCL-aggrssive-t3, ABCL-aggressive-t5) were used to impute the T5 dependent variables ASR-anxiety and ASR-aggressive behavior. Results were similar to the findings in our main analysis without multiple imputation. Therefore, we think that for the current study our analytic procedure was adequate and that it is not indicated/necessary to use other missing data techniques.
