# Supplementary material for: Attentional Bias for Cues Signaling Punishment and Reward in Adolescents: Cross-Sectional and Prognostic Associations with Symptoms of Anxiety and Behavioral Disorders
Source: J Abnorm Child Psychol. 2020 May 22;48(8):1007–21. doi: 10.1007/s10802-020-00654-3 (PMC7351843; doi:10.1007/s10802-020-00654-3)
Supplement: Supplementary file 1 — (DOCX 83.1 kb) [file 10802_2020_654_MOESM1_ESM.docx]

**Supplements**

| S1 Table with characteristics of SOT sample  *Number of participants in the low and high risk profile groups in the total TRAILS population (i.e., population) and in the focus cohort of participants who performed laboratory tasks* | | | | |
| --- | --- | --- | --- | --- |
|  |  | **Boys** | **Girls** | **Total** |
|  |  | **N** | **N** | **N** |
| Low risk (not A, B or C) | population  *focus cohort* | 462  *119* | 477  *123* | 939  *242* |
| Temperament (A) | population  *focus cohort* | 165  *53* | 138  *56* | 303  *109* |
| Parental psychopathology (B) | population  *focus cohort* | 142  *51* | 175  *52* | 317  *103* |
| Single-Parent family (C) | population  *focus cohort* | 79  *28* | 96  *38* | 175  *66* |
| A+B | population  *focus cohort* | 72  *33* | 66  *32* | 138  *65* |
| A+C | population  *focus cohort* | 41  *13* | 25  *10* | 66  *23* |
| B+C | population  *focus cohort* | 76  *31* | 99  *33* | 175  *64* |
| A+B+C | population  *focus cohort* | 57  *23* | 53  *20* | 110  *43* |
| Total | population  *focus cohort* | 1094  *351* | 1129  *364* | 2223  *715* |
| From *"* Reward-related attentional biases and adolescent substance use: The TRAILS study", by M.E*.* Van Hemel-Ruiter, P.J. De Jong, A. J. Oldehinkel, and B. Ostafin, 2013, Psychology of Addictive Behaviors, 27, Supplemental Material. *  *Note.* The selection criteria for high-risk profile group were as follows:   1. High-risk temperament: EATQ (Early Adolescent Temperament Questionnaire) Frustration ≥ 90^th^ percentile or EATQ Fear ≥ 90^th^ percentile or EATQ Effortful Control ≤ 10^th^ percentile. N_A_ = 617 (27.8%), 282 girls, 335 boys. 2. Parental psychopathology: at least one parent with severe psychopathology. N_B_ = 740 (33.3%), 393 girls, 347 boys 3. High environmental risk: at least one of both biological parents is not part of the family. N_C_ = 526 (23.7%), 273 girls, 253 boys. | | | | |

*no permission for reprinting was asked from the journal of Psychology of Addictive Behaviors, given APA guidelines stating: “

**3. Permission is Not Required for the Following:**

- A maximum of three figures or tables from a journal article or book chapter
- Single text extracts of less than 400 words
- Series of text extracts that total less than 800 words

No formal requests to APA or the author are required for the items in this clause.

<https://www.apa.org/about/contact/copyright/index#not-required>, visited on 18-12-2019

Permission was obtained from the first author of the paper M.E. van Hemel-Ruiter

B


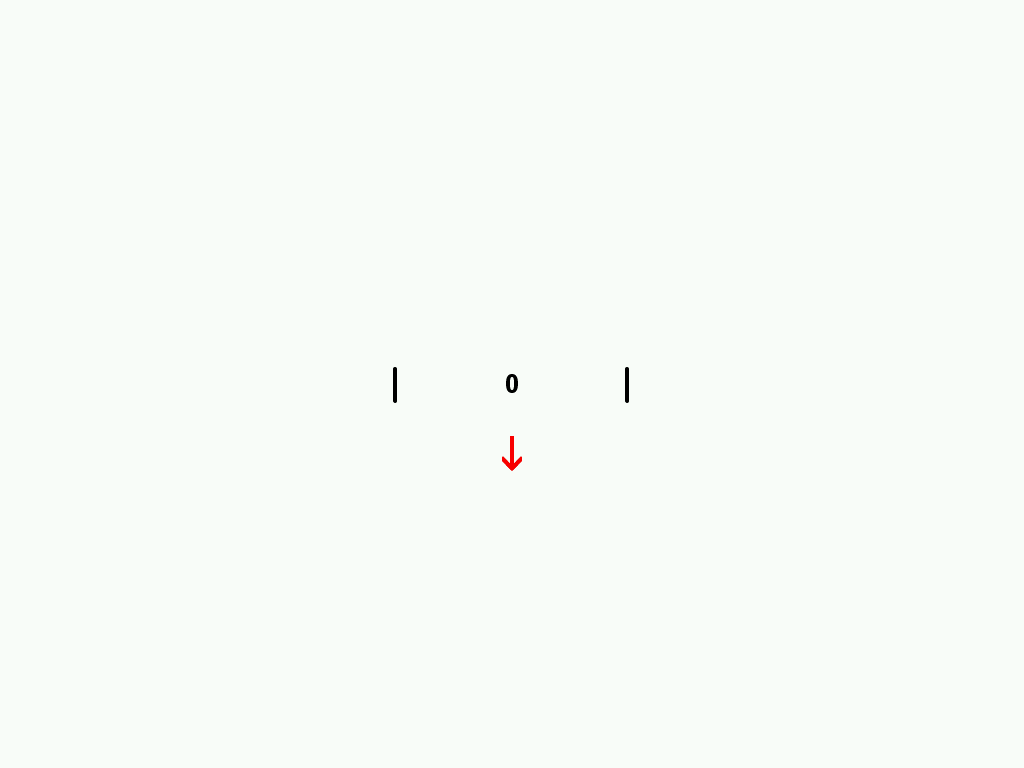


B


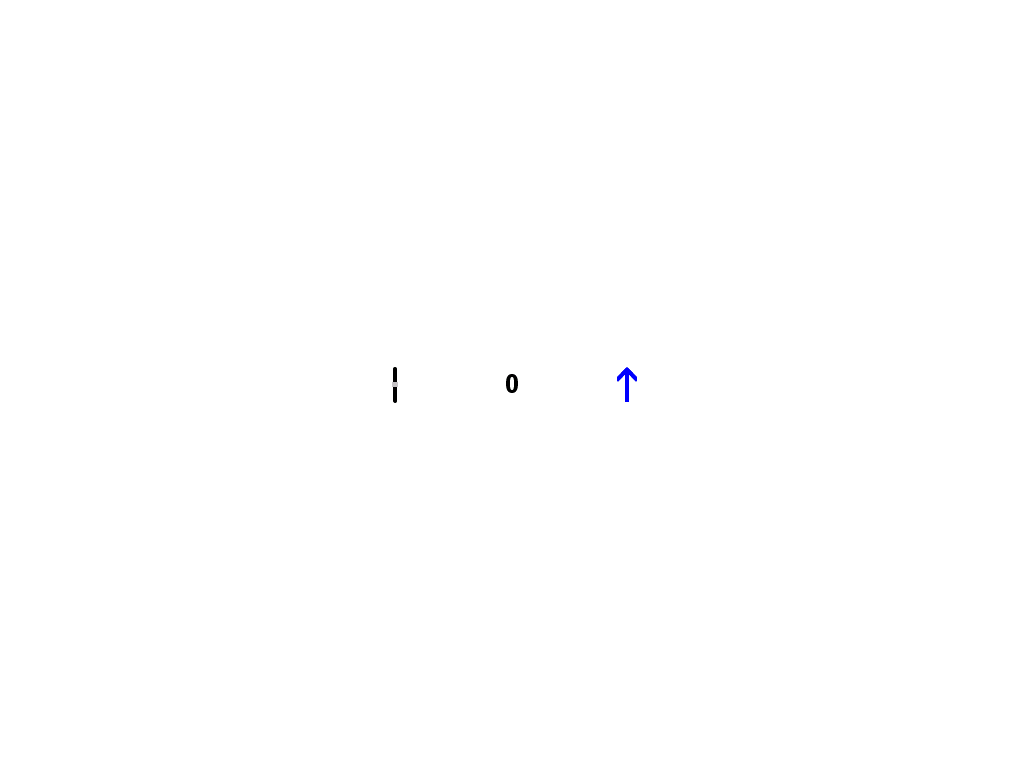


B


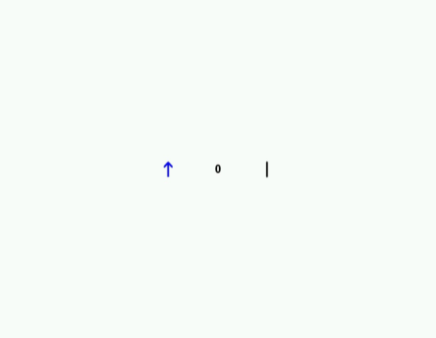


B


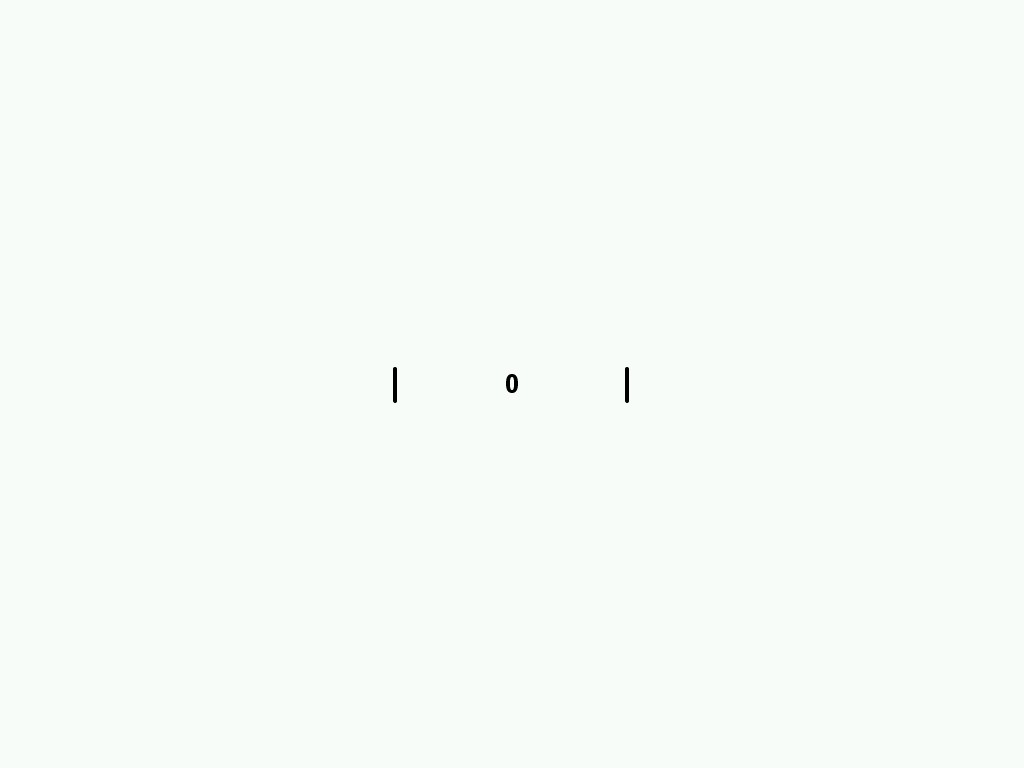


B


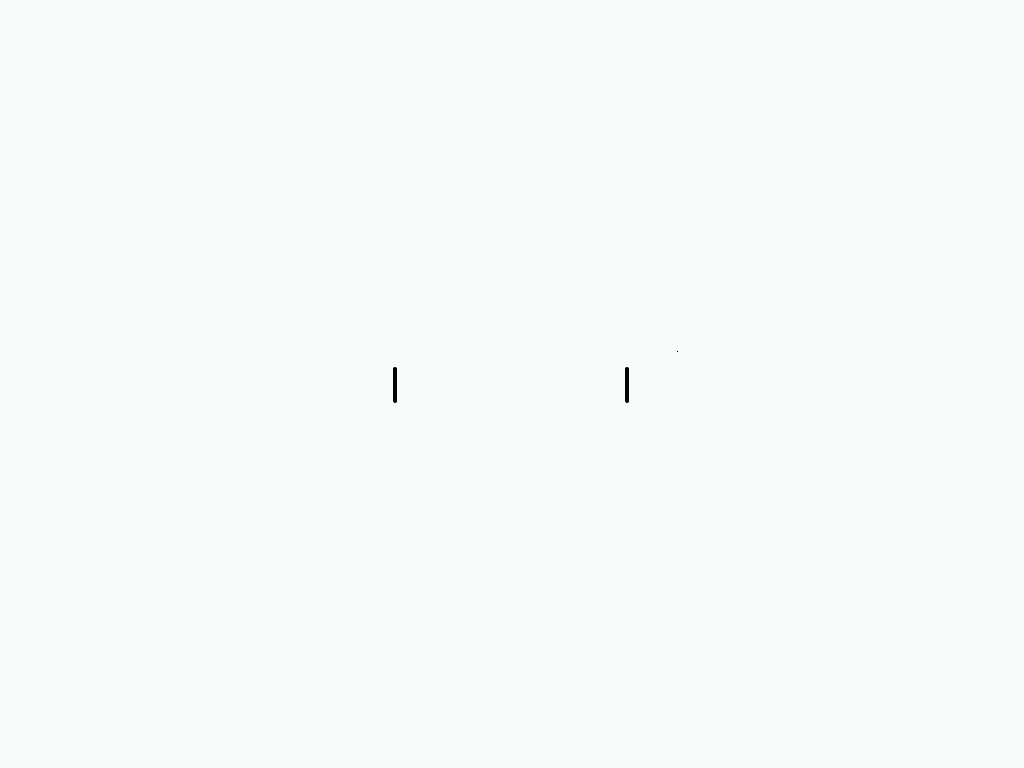


Press “B”

*S1 Figure* Example of screen-setup of the Spatial Orienting Task (SOT) - Example of blue cue, followed by target in the uncued location (i.e., hard target) with subsequent slow response (i.e., negative feedback). From *"* Reward-related attentional biases and adolescent substance use: The TRAILS study", by M.E*.* Van Hemel-Ruiter, P.J. De Jong, A. J. Oldehinkel, and B. Ostafin, 2013, Psychology of Addictive Behaviors, 27, Supplemental Material.*

*no permission for reprinting was asked from the journal of Psychology of Addictive Behaviors, given APA guidelines stating: “

**3. Permission is Not Required for the Following:**

- A maximum of three figures or tables from a journal article or book chapter
- Single text extracts of less than 400 words
- Series of text extracts that total less than 800 words

No formal requests to APA or the author are required for the items in this clause.

<https://www.apa.org/about/contact/copyright/index#not-required>, visited on 18-12-2019

Permission was obtained from the first author of the paper M.E. van Hemel-Ruiter
