# Supplementary material for: Attentional Bias for Cues Signaling Punishment and Reward in Adolescents: Cross-Sectional and Prognostic Associations with Symptoms of Anxiety and Behavioral Disorders
Source: J Abnorm Child Psychol. 2020 May 22;48(8):1007–21. doi: 10.1007/s10802-020-00654-3 (PMC7351843; doi:10.1007/s10802-020-00654-3)
Supplement: Supplementary file 2 — (DOCX 20 kb) [file 10802_2020_654_MOESM2_ESM.docx]

**Appendix A: Exploratory analyses**

***Step 3 Exploratory analyses:***

In order to see whether effects are game specific, we conducted the same analyses also with the cue validity effects with the other game type. Furthermore, we investigated whether the cue validity effects predicted change in anxiety and behavioral disorder symptoms from T3 to T5.

***Testing whether effects are game specific***

*Cross-sectional analyses:*

Anxiety symptoms (T3): No significant associations between the cue validity effects for cues signaling reward or non-reward with anxiety symptoms were found (see table 11).

| Table 11. regression model with anxiety (T3) and cue validity effects for reward and non-reward | | | | | | |
| --- | --- | --- | --- | --- | --- | --- |
| Dependent variable Anxiety T3 | *b* | *SE b* | *Beta* | *t* | *p* |  |
| Constant b0 | .425 | .021 |  | 19.81 | <.001 |  |
| CV-reward-short | 0.000 | .000 | -0.044 | -0.66 | .511 |  |
| CV-reward-long | 0.000 | .000 | -0.029 | -0.46 | .647 |  |
| CV-non-reward-short | 0.000 | .000 | 0.049 | 0.72 | .472 |  |
| CV-non-reward-long | 0.000 | .000 | -0.055 | -0.90 | .367 |  |
| *R^2­^_change . =._*006 | *F* =1.12 | *p =* .348 |  |  |  |  |
| *Note.* *n* = 696  Behavioral problems (T3): No significant associations between the cue validity effects for cues signaling punishment or non-punishment with behavioral problems were found (See table 12). | | | | | | |

| Table 12. regression model with behavioral problems (T3) and cue validity effects for punishment and non-punishment | | | | | | |
| --- | --- | --- | --- | --- | --- | --- |
| Dependent variable Behavioral problems T3 | *b* | *SE b* | *Beta* | *t* | *p* |  |
| Constant b0 | 0.321 | .016 |  | 20.68 | <.001 |  |
| CV-punishment-short | 0.000 | .000 | -0.083 | -1.22 | .222 |  |
| CV-punishment-long | 0.000 | .000 | 0.032 | 0.52 | .600 |  |
| CV-non-punishment-short | 0.000 | .000 | 0.020 | 0.29 | .770 |  |
| CV-non-punishment-long | 0.000 | .000 | -0.013 | -0.21 | .832 |  |
| *R^2­^_change . =._*004 | *F* =.63 | *p =* .645 |  |  |  |  |
| *Note.* *n* = 696 | | | | | | |

*Prospective analyses:*

Anxiety symptoms (T5): The cue validity effects for cues signaling reward or non-reward did not predict anxiety symptoms (see table 13).

| Table 13. regression model with anxiety (T5) and cue validity effects for reward and non-reward | | | | | | |
| --- | --- | --- | --- | --- | --- | --- |
| Dependent variable Anxiety T5 | *b* | *SE b* | *Beta* | *t* | *p* |  |
| Constant b0 | 0.394 | .028 |  | 13.86 | <.001 |  |
| CV-reward-short | 0.000 | .000 | -0.038 | -0.52 | .604 |  |
| CV-reward-long | 0.000 | .000 | -0.007 | -0.094 | .925 |  |
| CV-non-reward-short | 0.000 | .000 | 0.037 | 0.49 | .623 |  |
| CV-non-reward-long | 0.000 | .000 | -0.065 | -0.98 | .328 |  |
| *R^2­^_change . =._*005 | *F* =.82 | *p =* .516 |  |  |  |  |
| *Note.* *n* = 598 | | | | | | |

Behavioral problems (T5): The cue validity effects for cues signaling punishment or non-punishment did not predict behavioral problems (see table 14).

| Table 14. regression model with behavioral problems (T5) and cue validity effects for punishment and non-punishment | | | | | | |
| --- | --- | --- | --- | --- | --- | --- |
| Dependent variable Behavioral problems T5 | *b* | *SE b* | *Beta* | *t* | *p* |  |
| Constant b0 | 0.213 | .017 |  | 12.91 | <.001 |  |
| CV-punishment-short | 0.000 | .000 | 0.182 | 2.49 | .013 |  |
| CV-punishment-long | 0.000 | .000 | -0.029 | -0.44 | .660 |  |
| CV-non-punishment-short | 0.000 | .000 | -0.177 | -1.61 | .108 |  |
| CV-non-punishment-long | 0.000 | .000 | -0.107 | -1.56 | .120 |  |
| *R^2­^_change . =._*017 | *F* =2.63 | *p =* .033 |  |  |  |  |
| *Note.* *n* = 598 | | | | | | |

***Testing whether the cue validity effects predict change in anxiety and behavioral problems***

Anxiety symptoms (T5): The cue validity effects for cues signaling punishment or non-punishment did not predict change in anxiety symptoms (see table 15).

| Table 15. hierarchical regression model with anxiety (T5), anxiety (T3) and cue validity effects for punishment and non-punishment | | | | | | | |
| --- | --- | --- | --- | --- | --- | --- | --- |
| Dependent variable Anxiety T5 | | *b* | *SE b* | *Beta* | *t* | *p* |  |
| Step 1 | Constant b0 | .216 | 0.019 |  | 11.25 | <.001 |  |
|  | Anxiety T3 | .586 | 0.045 | 0.473 | 13.00 | <.001 |  |
|  |  |  |  |  |  |  |  |
| *R^2­^_change . =_*  .224 | *F* =168.86 | *P<.001* |  |  |  |  |  |
| Step 2 | Constant b0  Anxiety T3  CV-punishment-short  CV-punishment-long  CV-non-punishment-short  CV-non-punishment-long | .206  .574  .000  .000  .000  .000 | 0.026  0.045  0.000  0.000  0.000  0.000 | 0.464  0.118  -0.047  -0.046  -0.067 | 7.83  12.64  1.79  -0.81  -0.71  -1.09 | <.001  <.001  .073  .420  .476  .279 |  |
| *R^2­^_change . =._*006 | *F* =1.16 | *p=*.330 |  |  |  |  |  |
| *Note.* *n* = 587 | | | | | | | |

Behavioral problems: The cue validity effects for cues signaling reward or non-reward did not predict change in behavioral problems (see table 16).

| Table 16. hierarchical regression model with behavioral problems (T5), behavioral problems (T3) and cue validity effects for reward and non-reward | | | | | | | |
| --- | --- | --- | --- | --- | --- | --- | --- |
| Dependent variable Behavioral problems T5 | | *b* | *SE b* | *Beta* | *t* | *p* |  |
| Step 1 | Constant b0 | .099 | 0.014 |  | 6.95 | <.001 |  |
|  | Behavioral problems T3 | .373 | 0.037 | 0.388 | 10.21 | <.001 |  |
|  |  |  |  |  |  |  |  |
| *R^2­^_change . =._*151 | *F* =104.14 | *P<0.001* |  |  |  |  |  |
| Step 2 | Constant b0  Behavioral problems T3  CV-reward-short  CV-reward-long  CV-non-reward-short  CV-non-reward-long | .098  .368  .000  .000  .000  .000 | 0.020  0.037  0.000  0.000  0.000  0.000 | 0.384  -0.064  0.038  0.058  -0.074 | 4.90  10.03  -0.93  0.59  0.83  -1.20 | <.001  <.001  .351  .558  .407  .233 |  |
| *R^2­^_change . =._*004 | *F* =.70 | *p =* .592 |  |  |  |  |  |
| *Note.* *n* = 588 | | | | | | | |
