# Supplementary material for: Attentional Bias for Cues Signaling Punishment and Reward in Adolescents: Cross-Sectional and Prognostic Associations with Symptoms of Anxiety and Behavioral Disorders
Source: J Abnorm Child Psychol. 2020 May 22;48(8):1007–21. doi: 10.1007/s10802-020-00654-3 (PMC7351843; doi:10.1007/s10802-020-00654-3)
Supplement: Supplementary file 3 — (DOCX 46.6 kb) [file 10802_2020_654_MOESM3_ESM.docx]

**Appendix B: Results of preregistration**

Recently, more attention is being paid to the reliability of reaction time tasks. This also holds for the SOT task used in the current study.

The reliability of the cue validity effects can be improved by reducing the correlation between the components of the cue validitiy effect (RT of cued trials and RT of uncued trials). In order to achieve this, we subtracted the mean reaction time on the practice trials on either cued or uncued trials form their corresponding mean scores. As was found (in table 2), the reliability of the cue validity effect measures indeed improved after subtracting the individuals mean reaction time on practice trials. After this subtraction, the cue-validity effects were found to have adequate reliability.

Furthermore, by subtracting the individual’s mean reaction time on practice trial we also took individual differences in reaction times into account when calculating the cue validity effects, leading to a better indicator of the person’s cue validity effect.

Therefore, we think the adjusted version is a better way of calculating the cue validity effects and is therefore presented in our paper. Below you can find the results we found when strictly following our preregistration.

**Step 1:**

Bivariate correlations were calculated between the cue validity effects, anxiety symptoms and behavioral problems, see table 17. Table 17 shows that anxiety symptoms at T3 significantly correlated with anxiety symptoms at T5 and behavioral problems at T3 significantly correlated with behavioral problems at T5. Furthermore, anxiety symptoms and behavioral problems correlated significantly with each other. Furthermore, anxiety symptoms and behavioral problems at T5 correlated significantly with the cue validity effect for punishment in short cue delay trials.

|  | 1 | 2 | 3 | 4 | 5 | 6 | 7 | 8 | 9 | 10 | 11 | 12 | 13 |
| --- | --- | --- | --- | --- | --- | --- | --- | --- | --- | --- | --- | --- | --- |
| 1 Anxiety t3 (RCADS) | - |  |  |  |  |  |  |  |  |  |  |  |  |
| 2 Anxiety t3 (YSR) | .713* | - |  |  |  |  |  |  |  |  |  |  |  |
| 3 Behavioral problems t3 | .288 * | .271* | - |  |  |  |  |  |  |  |  |  |  |
| 4 Anxiety t5 | .467* | .473* | .224* | - |  |  |  |  |  |  |  |  |  |
| 5 Behavioral problems t5 | .385* | .337* | .388* | .641* | - |  |  |  |  |  |  |  |  |
| 6 CV-reward short | -.025 | .009 | -.048 | .037 | .024 | - |  |  |  |  |  |  |  |
| 7 CV-reward long | -.026 | -.007 | -.013 | .043 | .051 | .328* | - |  |  |  |  |  |  |
| 8 CV-nonreward short | .007 | .057 | -.015 | .062 | .058 | .553* | .390* | - |  |  |  |  |  |
| 9 CV-nonreward long | -.039 | -.027 | -.032 | .020 | .019 | .174* | .421* | .311* | - |  |  |  |  |
| 10 CV-punishment short | .042 | .065 | -.031 | .108* | .104* | .542* | .344* | .577* | .214* | - |  |  |  |
| 11 CV-punishment long | -.076 | -.025 | .018 | -.012 | .019 | .167* | .352* | .272* | .373* | .272* | - |  |  |
| 12 CV-nonpunishment short | -.036 | .006 | -.002 | .017 | .006 | .516* | .253* | .481* | .185* | .513* | .210* | - |  |
| 13 CV-nonpunishment long | -.039 | .008 | .001 | -.026 | -.017 | .287* | .402* | .292* | .326* | .279* | .389* | .271* | - |

Table 17: Bivariate correlations of cue validity effects with internalizing and behavioral problems at T3 and T5.

Note: correlations between t3 variables and CV variables are based on a sample size of n = 696, correlations with t5 variables are based on a sample size of n = 586. *p<0.01

**Step 2 Main analyses**

We performed multiple regression analyses, results of the analyses are presented in the tables below. For the main analyses the cue validity effects from the losing games were used as predictors for anxiety symptoms, whereas the cue validity effects from the winning games were used as predictor variables for behavioral problems.

*Cross-sectional analyses:*

1. Anxiety symptoms (T3): No significant associations between the cue validity effects for cues signaling punishment or non-punishment with anxiety were found.

| Dependent variable anxiety T3 | *b* | *SE b* | *Beta* | *t* | *p* |
| --- | --- | --- | --- | --- | --- |
| Constant b0 | .425 | .024 |  | 17.53 | <.001 |
| CV-punishment-short | 0.000 | .000 | 0.105 | 2.32 | .021 |
| CV-punishment-long | 0.000 | .000 | -0.083 | -2.00 | .046 |
| CV-non-punishment-short | 0.000 | .000 | -0.067 | -1.50 | .134 |
| CV-non-punishment-long | 0.000 | .000 | -0.018 | -0.43 | .669 |
| *R^2­^_change . = ._*014 |  |  |  |  |  |

Table 18: regression model with anxiety (T3) and cue validity effects for punishment and nonpunishment

N = 696

1. Behavioral problems (T3) : No significant associations between the cue validity effects for cues signaling reward or non-reward with behavioral problems were found (see table 19).

Table 19: regression model with behavioral problems (T3) and cue validity effects for reward and nonreward

| Dependent variable Behavioral problems T3 | *b* | *SE b* | *Beta* | *t* | *p* |
| --- | --- | --- | --- | --- | --- |
| Constant b0 | 0.327 | .020 |  | 16.13 | <.001 |
| CV-reward-short | 0.000 | .000 | - 0.059 | -1.28 | .201 |
| CV-reward-long | 0.000 | .000 | 0.011 | 0.24 | .807 |
|  |  |  |  |  |  |
| CV-non-reward-short | 0.000 | .000 | 0.024 | -0.50 | .615 |
| CV-non-reward-long | 0.000 | .000 | -0.034 | -0.80 | .423 |
| *R^2­^_change . = ._*003 |  |  |  |  |  |

N= 696

*Prospective analyses:*

1. Anxiety symptoms (T5): It was found that having a larger cue validity effect for punishing cues with short cue delay predicted higher anxiety symptoms (see table 20).

Table 20: regression model with anxiety (T5) and cue validity effects for punishment and nonpunishment

| Dependent variable anxiety T5 | *b* | *SE b* | *Beta* | *t* | *p* |
| --- | --- | --- | --- | --- | --- |
| Constant b0 | 0.360 | .033 |  | 11.06 | <.001 |
| CV-punishment-short | 0.001 | .000 | 0.150 | 3.08 | .002* |
| CV-punishment-long | 0.000 | .000 | -0.025 | -0.56 | .576 |
| CV-non-punishment-short | 0.000 | .000 | -0.041 | -0.86 | .393 |
| CV-non-punishment-long | 0.000 | .000 | -0.048 | -1.06 | .288 |
| *R^2­^_change . = ._*017 |  |  |  |  |  |

N = 598 *significant at p < .0125

1. Behavioral problems: The cue validity effects for cues signaling reward or non-reward did not predict behavioral problems (see table 21).

| Dependent variable Behavioral problems T5 | *b* | *SE b* | *Beta* | *t* | *p* |
| --- | --- | --- | --- | --- | --- |
| Constant b0 | 0.199 | .021 |  | 9.28 | <.001 |
| CV-reward-short | 0.000 | .000 | -0.020 | -0.39 | .694 |
| CV-reward-long | 0.000 | .000 | 0.041 | 0.86 | .389 |
| CV-non-reward-short | 0.000 | .000 | 0.058 | 1.12 | .265 |
| CV-non-reward-long | 0.000 | .000 | -0.013 | -0.29 | .774 |
| *R^2­^_change . = ._*005 |  |  |  |  |  |

Table 21: regression model with behavioral problems (T5) and cue validity effects for reward and nonreward

N= 598

**Step 3 Exploratory analyses:**

***Testing whether effects are game specific***

We also conducted these regression analyses with cue validity effects from the winning games for anxiety symptoms and cue validity effects from the losing games for behavioral problems to check the specificity of the effects on winning and losing games.

*Cross-sectional analyses:*

1. Anxiety symptoms (T3): No significant associations between the cue validity effects for cues signaling reward or non-reward with anxiety symptoms were found (see table 22).

Table 22: regression model with anxiety (T3) and cue validity effects for reward and nonreward

| Dependent variable Anxiety T3 | *b* | *SE b* | *Beta* | *t* | *p* |
| --- | --- | --- | --- | --- | --- |
| Constant b0 | .432 | .025 |  | 17.10 | <.001 |
| CV-reward-short | 0.000 | .000 | -0.039 | -0.85 | .397 |
| CV-reward-long | 0.000 | .000 | -0.015 | -0.33 | .741 |
| CV-non-reward-short | 0.000 | .000 | 0.047 | .97 | .333 |
| CV-non-reward-long | 0.000 | .000 | -0.041 | -0.95 | .341 |
| *R^2­^_change . = ._*003 |  |  |  |  |  |

N = 696

1. Behavioral problems (T3): No significant associations between the cue validity effects for cues signaling punishment or non-punishment with behavioral problems were found (see table 23).

Table 23: regression model with behavioral problems (T3) and cue validity effects for punishment and nonpunishment

| Dependent variable Behavioral problems T3 | *b* | *SE b* | *Beta* | *t* | *p* |
| --- | --- | --- | --- | --- | --- |
| Constant b0 | 0.319 | .020 |  | 16.32 | <.001 |
| CV-punishment-short | 0.000 | .000 | -0.046 | -1.02 | .309 |
| CV-punishment-long | 0.000 | .000 | 0.028 | 0.67 | .504 |
| CV-non-punishment-short | 0.000 | .000 | 0.016 | 0.37 | .713 |
| CV-non-punishment-long | 0.000 | .000 | -0.002 | -0.042 | .967 |
| *R^2­^_change . = ._*002 |  |  |  |  |  |

N= 696

*Prospective analyses:*

1. Anxiety symptoms (T5): The cue validity effects for cues signaling reward or non-reward did not predict anxiety symptoms (see table 24).

Table 24: regression model with anxiety (T5) and cue validity effects for reward and nonreward

| Dependent variable Anxiety T5 | *b* | *SE b* | *Beta* | *t* | *p* |
| --- | --- | --- | --- | --- | --- |
| Constant b0 | 0.365 | .033 |  | 10.90 | <.001 |
| CV-reward-short | 0.000 | .000 | -0.002 | -0.04 | .968 |
| CV-reward-long | 0.000 | .000 | 0.026 | 0.54 | .587 |
| CV-non-reward-short | 0.000 | .000 | 0.056 | 1.07 | .286 |
| CV-non-reward-long | 0.000 | .000 | -0.008 | -0.17 | .864 |
| *R^2­^_change . = ._*004 |  |  |  |  |  |

N = 598

1. Behavioral problems (T5): It was found that having a larger cue validity effect for punishing cues with short cue delay predicted more behavioral problems (see table 25).

Table 25: regression model with behavioral problems (t5) and cue validity effects for punishment and nonpunishment

| Dependent variable Behavioral problems T5 | *b* | *SE b* | *Beta* | *t* | *p* |
| --- | --- | --- | --- | --- | --- |
| Constant b0 | 0.198 | .021 |  | 9.49 | <.001 |
| CV-punishment-short | 0.000 | .000 | 0.143 | 2.94 | .003* |
| CV-punishment-long | 0.000 | .000 | 0.010 | 0.22 | .825 |
| CV-non-punishment-short | 0.000 | .000 | -0.056 | -1.16 | .247 |
| CV-non-punishment-long | 0.000 | .000 | -0.046 | -1.02 | .309 |
| *R^2­^_change . = ._*002 |  |  |  |  |  |

N = 598 *significant at p < .0125

***Testing whether the cue validity effects predict change in anxiety and externalizing problems***

We also conducted these regression analyses with the cue validity effects from the losing games on anxiety symptoms at T5, when controlling for anxiety symptoms at T3 and with the cue validity effects of the winning games on behavioral problems at T5, when controlling for behavioral problems at T3.

1. Anxiety symptoms (T5): The cue validity effects for cues signaling punishment or non-punishment did not predict change in anxiety symptoms (see table 26).

| Table 26. hierarchical regression model with anxiety (T5), anxiety (T3) and cue validity effects for punishment and non-punishment | | | | | | | | | |
| --- | --- | --- | --- | --- | --- | --- | --- | --- | --- |
| Dependent variable Anxiety T5 | | | *b* | | *SE b* | *Beta* | *t* | *p* |  |
| Step 1 | Constant b0 | | 0.216 | | .019 |  | 11.25 | <.001 |  |
|  | Anxiety T3 | | 0.586 | | .045 | 0.473 | 13.00 | <.001 |  |
|  |  | |  | |  |  |  |  |  |
| *R^2­^_change . =_*.110 | |  | |  |  |  |  |  |  |
| Step 2 | Constant b0  Anxiety T3  CV-punishment-short  CV-punishment-long  CV-non-punishment-short  CV-non-punishment-long | | 0.195  0.577  0.000  0.000  0.000  0.000 | | .031  .045  .000  .000  .000  .000 | 0.466  0.089  -0.017  -0.021  -0.032 | 6.21  12.74  2.03  -0.42  -0.49  -0.79 | <.001  <.001  .043  .673  .626  .429 |  |
| *R^2­^_change =._*007 |  | |  | |  |  |  |  |  |
| *Note.* *n* = 588 | | | | | | | | | |

1. Behavioral problems: The cue validity effects for cues signaling reward or non-reward did not predict change in behavioral problems (see table 27).

| Table 27. hierarchical regression model with behavioral problems (T5), behavioral problems (T3) and cue validity effects for reward and non-reward | | | | | | | |
| --- | --- | --- | --- | --- | --- | --- | --- |
| Dependent variable Behavioral problems T5 | | *b* | *SE b* | *Beta* | *t* | *p* |  |
| Step 1 | Constant b0 | 0.099 | 0.014 |  | 6.95 | <.001 |  |
|  | Behavioral problems T3 | 0.373 | 0.037 | 0.388 | 10.21 | <.001 |  |
|  |  |  |  |  |  |  |  |
| Step 2 | Constant b0  Behavioral problems T3  CV-reward-short  CV-reward-long  CV-non-reward-short  CV-non-reward-long | 0.075  0.376  0.000  0.000  0.000  0.000 | .023  .037  .000  .000  .000  .000 | 0.391  -0.013  0.055  0.064  -0.010 | 3.29  10.25  -0.29  1.24  1.32  -0.24 | .001  <.001  .775  .216  .189  .811 |  |
| *Note.* *n* = 588 | | | | | | | |

**Step 4 (only when necessary based on previous results)**

We did not find effects in both the losing and winning games on either behavioral problems or anxiety symptoms. Therefore, this step was not conducted.
